# Doing-together with words: the sequential unfolding of a moment of meeting in a psychoanalytic therapy session

**DOI:** 10.3389/fpsyg.2023.1205500

**Published:** 2023-12-07

**Authors:** Marcos Herrera, Andrea Ugarte, Gabriela Vásquez-Torres, Kene M. Durand, Miguel Sánchez

**Affiliations:** ^1^Department of Humanities, Pontifical Catholic University of Peru, Lima, Peru; ^2^Department of Psychology, Pontifical Catholic University of Peru, Lima, Peru; ^3^Escuela de Posgrado, Pontifical Catholic University of Peru, Lima, Peru

**Keywords:** conversation analysis, psychotherapy interaction, psychoanalysis, moments of meeting, therapeutic relationship, transformative sequence, transformation of relation, linguistic pragmatics

## Abstract

Changes in psychoanalytic therapy have been traditionally attributed to self-knowledge (insight) in the client, provided by the therapist’s interpretations. In recent years there has been growing realization that such changes can also be the consequence of the development of new forms of relatedness through client–therapist interaction, particularly through special intersubjective moments called moments of meeting. Drawing on the methods and findings of Conversation Analysis about the sequential organization of psychotherapeutic interaction, this single-case study examines the unfolding of a moment of meeting in the final session of a brief psychoanalytic therapy in Peru (in Spanish) with a female client victim of domestic violence. Our analysis shows that the moment of meeting, which resolves a challenge to the intersubjective relationship posed by a now moment, comes about interactionally through a sequentially accomplished shared practice of co-animation. In this sequence the client, who had previously assumed a passive role, exercises her own agency to assume an active role, which the therapist ratifies through his response. In this way, a momentary but significant transformation in the here-and-now relationship between client and therapist occurs. Thus, our analysis contributes to the understanding of how a transformation of relation—the transitory emergence of a new form of relatedness—can take place in and through sequentially organized talk and action in psychotherapy. Our study also sheds light on the role of language in moments of meeting, as the moment of meeting in our segment does not occur in parallel with the exchange of linguistic utterances between client and therapist, but through the exchange of such linguistic utterances and through the sequence of actions carried out by that exchange. In this way, the sequential doing-together with words leads to a moment of meeting, bringing about change, at least momentarily, in the implicit ways-of-being-with-others of the client.

## Introduction

In an influential article, Peräkylä (2019) points to a double task of Conversation Analysis (CA) research on psychotherapy: (1) to investigate how the machinery of interaction (the organization of sequences of action) is adapted for the institutional goals of therapists and clients; and (2) to investigate how therapeutically relevant change takes place in and through these action sequences. Regarding (1), he proposes a *general model of sequential organization of psychotherapy interaction* as a useful heuristic for researchers to identify sequential relations in their data. It consists of a *Prior Action* (PA); an initiating *Target Action* (TA), which is the focus of the analysis; a responding action or *Response* (RE); and a *Third Position Action* (TP) closing the exchange (Peräkylä, 2019; Muntigl, 2020). Regarding (2), Peräkylä points out that transformation of experience plays a crucial role in psychotherapy process; drawing on the CA principle of *nextness* (Schegloff, 2007), he proposes that such a sequence of adjacent conversational turns can be considered a vehicle for transformation of experience: “‘Nextness”’ of any turn at talk makes it inevitable that the current speaker will orient him/herself to the experience embodied in the prior turn.” (Peräkylä, 2019, p. 266). He further distinguishes three main, overlapping domains for this transformation of experience: “three psychosocial processes that take place through the sequentially organized talk and action: transformation of referents, transformation of emotion, and transformation of relation.” (Peräkylä, 2019, p. 266).

Regarding the transformation of relation, Peräkylä states that psychotherapeutic encounters document, reproduce, and renew (moment by moment) the particular socioemotional relation between that particular therapist and that particular client. CA research on this topic includes key relational phenomena like *agreement* and *disagreement* or *resistance*, *affiliation* and *disaffiliation*, and the *epistemic relation* between participants (Voutilainen et al., 2010; Muntigl et al., 2012; Scarvaglieri, 2020; Guxholli et al., 2021): “These and other aspects of the momentary relation get transformed through sequentially organized actions.” (Peräkylä, 2019, p. 271).

We can point out an additional, significant aspect of the momentary relation that can get transformed through sequentially organized actions: the change in the here-and-now relationship between client and therapist, as manifested in the interaction between them. From the perspective of contemporary relational psychoanalysis, such changes are construed as changes in “relatedness” (Mitchell, 1988; Stern, 2009). This refers to the basic human capacity to form relations with others, and also to the particular relational patterns shaping a person’s interpersonal life; they are mostly unconscious and derive from early relations with our caregivers in infancy.

The relevance of relatedness for psychoanalytic therapy has been prompted by the “relational” or “intersubjective” turn in psychoanalysis (Mitchell and Aron, 1999; Schwartz, 2012). From a more traditional perspective, changes during the psychoanalytic process have been attributed to self-knowledge (insight) in the client, provided by the therapist’s veridical interpretations, i.e., verbal statements “corresponding” to the client’s conscious or unconscious subjectivity. Thus, in his account of the “classic” technique of *ego psychology*, Wallerstein (2002) identifies the assumption that “the analyst’s veridical interpretations, properly reinforced through the process of working through” were “the necessary and sufficient road to insight, change, and cure” (p. 141). In recent years, however, there has been growing realization that changes within psychoanalytic therapy are also consequences of “something more than interpretation” (in the sense of making the unconscious conscious), and that this “something more” is linked to intersubjective interactional processes (Stern et al., 1998). In particular, contemporary relational psychoanalysis attributes changes during the therapeutic process to the development of new forms of relatedness through client–therapist interaction (Mitchell, 1997; Stern, 2009). From this perspective, the therapeutic action occurs not only through the content of the therapist’s verbal interpretations but also through the interaction unfolding between therapist and client.

One of the more influential theoretical frameworks accounting for such changes in relatedness—i.e., changes in the here-and-now relationship between client and therapist—comes from the work by D. Stern and the Boston Change Process Study Group (BCPSG). Based on studies of early mother–infant interaction, they claim that therapeutic changes result from the influence of interactional intersubjective processes between therapist and client on the client’s implicit relational knowing. Thus, special intersubjective moments can not only reorganize the relationship between the interactants but also, more importantly, change the client’s *implicit procedural knowledge*—his/her *ways-of-being-with-others* (Stern et al., 1998; Stern, 2004). Standing out amongst such key intersubjective moments are *moments of meeting*. In what follows we draw mainly on the presentation of this concept in Stern’s (2004) influential book. He considers them a special kind of *present moments*, which are small and momentary events that build up our conscious experience. Moments of meeting are intersubjective present moments, because they are shared between two people. Although they also occur in everyday life, they are crucial moments for change in psychotherapy. In the first chapter of his book, Stern (2004) offers a beautiful and touching example of such an event. A therapist used to shake hands with his clients at the end of the session as a goodbye gesture. One day, the client narrated a moving sequence of events that affected both him and the therapist deeply. At the end of the session, during the regular goodbye handshake, the therapist laid his left hand on the client’s right hand, which he was holding already. This resulted in a two-handed shake: “They looked at each other. Nothing was said. The whole thing lasted several seconds. It was not talked about in subsequent sessions either. Yet, the relationship had shifted on its axis” (Stern, 2004, p. 19).

In Stern’s (2004) theoretical account, moments of meeting follow other important intersubjective moments called *now moments*. These interpersonal events challenge the ongoing relation between client and therapist, threatening the intersubjective field and creating a crisis that needs resolution, which can potentially be provided by the moments of meeting. To illustrate, we use another example from Stern (2004, pp. 166–169) concerning a female client in psychoanalytic treatment with a female therapist. During one session, after lying for some time on the couch, the client suddenly said, “I want to sit and look at your face.” She then sat up and faced her therapist, who was sitting behind the couch. Client and therapist looked at each other in silence, puzzled. This was a now moment that threatened the intersubjective field, testing the therapist and the therapy. Spontaneously, the therapist smiled at her client, lightly tilted her head, and said, “Hello.” They then continued to look at each other for several seconds until the client laid back on the couch and continued talking, doing her analytic work but now more profoundly. This was a moment of meeting, in which the participants seek “intersubjective ‘fittedness”’ (Stern, 2004, p. 168). Contributions by the therapist that can lead to moments of meeting are usually authentic responses finely tailored to the momentary local situation. They are spontaneous and personal, not just neutral and technical responses. Stern stresses that moments of meeting do not need to be verbalized to effect change. They would mainly result from interactions at an implicit level, parallel to the exchange of language at the explicit level. We will return to this issue in the Discussion.

Recent research on psychoanalytic psychotherapy has been sensitive to the relational or intersubjective turn in psychoanalysis, assuming the dyadic and interactional nature of psychoanalytic therapy (Bohleber, 2013; Altimir and Jiménez, 2020). Interest is increasing in a microscopic inquiry of the interaction in relevant episodes of therapy sessions (Krause and Altimir, 2016). In that regard, CA is a convenient method to investigate in detail this relational aspect of the psychotherapeutic process as manifested in the sequential exchange between client and therapist. It allows us to examine how significant moments in the psychotherapeutic process come about interactionally. CA has been successfully applied to study psychotherapy interaction (Peräkylä, 2008, 2013; Voutilainen et al., 2011; Buchholz and Kächele, 2013; Guxholli et al., 2021; Peräkylä and Buchholz, 2021). One main result of this research is that to understand therapeutic interaction, we need to examine its sequential organization (Peräkylä, 2013, 2019).

Our paper draws on the methods and findings of CA, particularly Peräkylä’s (2019) sequential model, to present a single-case analysis of an episode from the final session of a brief psychoanalytic therapy in Peru with a female client who has experienced domestic violence. We examine how a moment of meeting comes about interactionally and how a momentary change in the here-and-now relationship between client and therapist takes place through sequentially organized talk and action during that moment of meeting.

## Data and methods

We focus on an episode in the last session of a brief psychoanalytic psychotherapy. The data are sourced from the Grupo de Investigación en Psicoanálisis (Research Group on Psychoanalysis) of the Pontifical Catholic University of Peru, in the context of the research project *Dialogic Moments of Meeting. An Application of Conversation Analysis to Sessions of Brief Psychoanalytic Psychotherapy*, supported by a Grant of the Research Committee of the International Psychoanalytic Association in cooperation with the Pontifical Catholic University of Peru, 2022–2023. Our study received ethical approval from the Ethics Committee of the Pontifical Catholic University of Peru. The participants gave written informed consent for the use of the data for research and publication.

The client is a 37-year-old woman, to whom we give the pseudonym “Luz.” She is a migrant from a rural area who lives in Lima, the capital city of Peru, in an economically precarious situation. She has been a victim of domestic violence and presented symptoms of depression and anxiety, along with signs of post-traumatic stress disorder. The psychotherapeutic treatment is given in a public institution that helps low-income women. The therapist is a 31-year-old male clinical psychologist who has received training in psychoanalytic psychotherapy. Only an audio recording of the session was feasible. The therapy comprised 12 sessions of Brief Dynamic Interpersonal Therapy (DIT), a focal psychodynamic psychotherapy centered on the client’s relationships as they are related to current life problems and symptoms of depression or anxiety (Lemma et al., 2011).

Whilst CA commonly draws on collections of multiple instances of an interaction phenomenon, previous research has used analysis of single episodes of interaction to apply prior knowledge on the organization of a domain of talk-in-interaction to illuminate a specific segment of talk (Schegloff, 1987; Whalen et al., 1988). In CA studies of psychotherapy, for instance, a single-case analysis has been used to illustrate how client and therapist manage impasses to emotional exploration, mapping the clinically relevant trajectory through which they can successfully secure extended and intense emotional work (Muntigl, 2020).

We used methods of CA for the transcription and for the analysis of the session. Considered among qualitative research methods in psychology (Willig and Stainton-Rogers, 2008), CA facilitates the investigation of talk-in-interaction based on careful empirical examination of detailed transcriptions of interactional phenomena (Schegloff, 2007; Ten Have, 2007; Wilkinson and Kitzinger, 2008; Stivers, 2013; Raymond and Olguín, 2022). Therefore, it is a convenient method for analyzing the relational aspect of the psychotherapeutic process as manifested in client–therapist exchanges. For instance, the client–therapist relationship has been investigated from an interactionist perspective using CA to analyze the interaction between therapist and client (Scarvaglieri, 2020).

The chosen segment attracted our attention because it revealed a remarkable change in the here-and-now relationship between client and therapist. We then applied CA concepts and tools to analyze the interactional unfolding of that particular change. During our analysis, we noticed that this episode showed some features of Stern’s (2004) “moments of meeting.” A more careful study of this theoretical approach (Stern et al., 1998; Stern, 2004) allowed us to analyze our segment applying categories belonging to that framework, like *now moment* and *moment of meeting*. Our next goal was to bring both approaches together in the analysis of the segment, in order to provide an account of how a moment of meeting comes about interactionally. First, we examined the segment using CA’s activity known as *data sessions* (Ten Have, 2007): the group of researchers analyzed in detail the transcript and the audio recording of the segment, focusing on the sequential relations between turns. Second, we shared our data with two other groups of researchers in online data sessions, the first in the field of CA applied to conversational data in Spanish, the Seminario Permanente de Análisis de la Conversación (SPAC) (Ongoing Seminar in Conversation Analysis), and the second in the field of CA research on psychotherapy, the team of Prof. Anssi Peräkylä (University of Helsinki). Third, we gathered and systematized observations by participants in both online data sessions. Fourth, based on this systematization, we outlined a sequential interpretation of the whole segment. Fifth, drawing on Stern’s (2004) theoretical framework, we tried to identify the now moment and the moment of meeting in our segment. Finally, we applied Peräkylä’s (2019) sequential model to illustrate how a change in the here-and-now relationship between client and therapist—a moment of meeting—unfolds step by step in the interaction between therapist and client in that segment.

## Results

The following Extract shows the transcription of the audio recording of an episode during the last session of the therapy. We have used CA jeffersonian transcription conventions (Jefferson, 2004; Ten Have, 2007; Raymond and Olguín, 2022; see Supplementary Appendix). In accordance with the principles of DIT, one main goal of the treatment was to help this client become aware of an interpersonal-affective focus (a representation of self-in-relation-to-another) whereby she perceived herself as a submissive, dependent woman and other people as aggressive and dominant, generating a pervasive relational pattern of passivity toward others. Accordingly, one central objective of the therapist’s verbal statements was to foster the client’s agency, which is especially relevant for victims of domestic violence (Hirigoyen, 2006). In this last session, the therapist is trying to accomplish an interactional project following the DIT guidelines for terminating therapy: give the client an outline of the main results, highlight her resources, and address the end of treatment.

In our analysis of this episode, we interweave both CA and Stern’s (2004) theoretical framework. In that regard, Buchholz (2018) has pointed out the contribution that CA can make to a detailed interactional account of moments of meeting in psychotherapy. Accordingly, we apply CA, particularly Peräkylä’s (2019) sequential model, with two goals. First, to examine how a moment of meeting, which resolves a challenge to the intersubjective relationship posed by a now moment, comes about interactionally in this episode. Second, to examine how a transitory transformation in the here-and-now relationship between client and therapist takes place interactionally during that moment of meeting.

Next, we present our single-case analysis of this episode. We divide the segment into five sections. As our analysis will show, the now moment occurs in section 2 (12–17) and the moment of meeting in section 4 (24–30).

### Section 1 (01–11)

The interaction business of this episode involves the management of the ending of the therapeutic relationship and the impending separation. In accordance with this, the therapist produces two long turns (01–07) and (09–11), in which he points to the client’s agency and autonomy, which should enable her to carry on the work by herself. The client contributes just one single turn (06) in this section, uttering the word “yes” in a rather low voice. The client–therapist interaction in this first section exhibits some features characteristic of their exchange during most of the session up to this point: the therapist has the turn most of the time and talks in a didactic style to the client, who limits herself to giving weak signals of acknowledgment. We notice that their interaction displays an implicit relational pattern where the therapist has an active role while the client remains passive. This is at odds with the explicit content of the therapist’s contributions during the treatment, whose goal is to foster the client’s agency and to change her pervasive pattern of displaying a passive attitude toward others. Moreover, it seems that this very relational pattern that the therapy aims to change is shaping the here-and-now relationship between client and therapist and their interaction.

### Section 2 (12–17)

After a gap of 7.4 s (12), the client utters the interjection “ay” (which in Spanish conveys pain) and then sighs (13). Next, she says that she wants God to give her strength and not to let her go (15–16); after that, both client and therapist are silent (17). We notice that the client does not embrace what the therapist has said, thus not affiliating herself with his stance. Through her expression of pain and invocation of an external force (God) to remain with her, she projects a vulnerable position dependent on God’s continued assistance. It is striking that in the final session, where the therapist is letting go of her, the client confronts him with the hope that God will not abandon her. Her appeal to God for strength fundamentally challenges the therapist’s assertion that she can carry on the work by herself, strongly refuting the agency he credits her with.

This powerful situation constitutes the now moment, where a challenge to the intersubjective relationship comes about, unleashing a crisis that needs resolution through a moment of meeting (Stern, 2004). As we will see below, our segment contains two attempts to achieve this moment of meeting. The first one in section 3 (18–23) fails but the second one in section 4 (24–30) succeeds.

### Section 3 (18–23)

After a gap of 5.8 s (17) we have a new turn by the therapist (18–19). He tells her that although she feels helpless and vulnerable, she actually has strength of which she is unaware. What kind of action is performed by this turn? CA research has shown that there are two important actions usually performed by therapists when responding to things that the client has said. These are *formulations* and *interpretations* (Antaki, 2008). The action performed by this turn does not seem to be a formulation, which would aim to put into words the content of the client’s previous turn but from her own perspective (Antaki, 2008). It seems to be rather an interpretation, because its design displays that it presents the therapist’s understanding of the client’s experience from his own perspective (Bercelli et al., 2008; Peräkylä and Antaki, 2008; Peräkylä, 2013). We notice that the therapist introduces his interpretation in this turn with the expression “To this I would add.” On the one hand, these words aim to prevent the client perceiving this turn as an attempted topic shift (Jefferson, 1993) by purportedly expanding on the topic she introduced previously. On the other hand, although the interpretation challenges the client’s stance and self-presentation, as most interpretations do (Peräkylä and Antaki, 2008; Deppermann et al., 2020), it does not confront her feelings of vulnerability. In both cases, it contributes to preserving affiliation.

**Figure:**
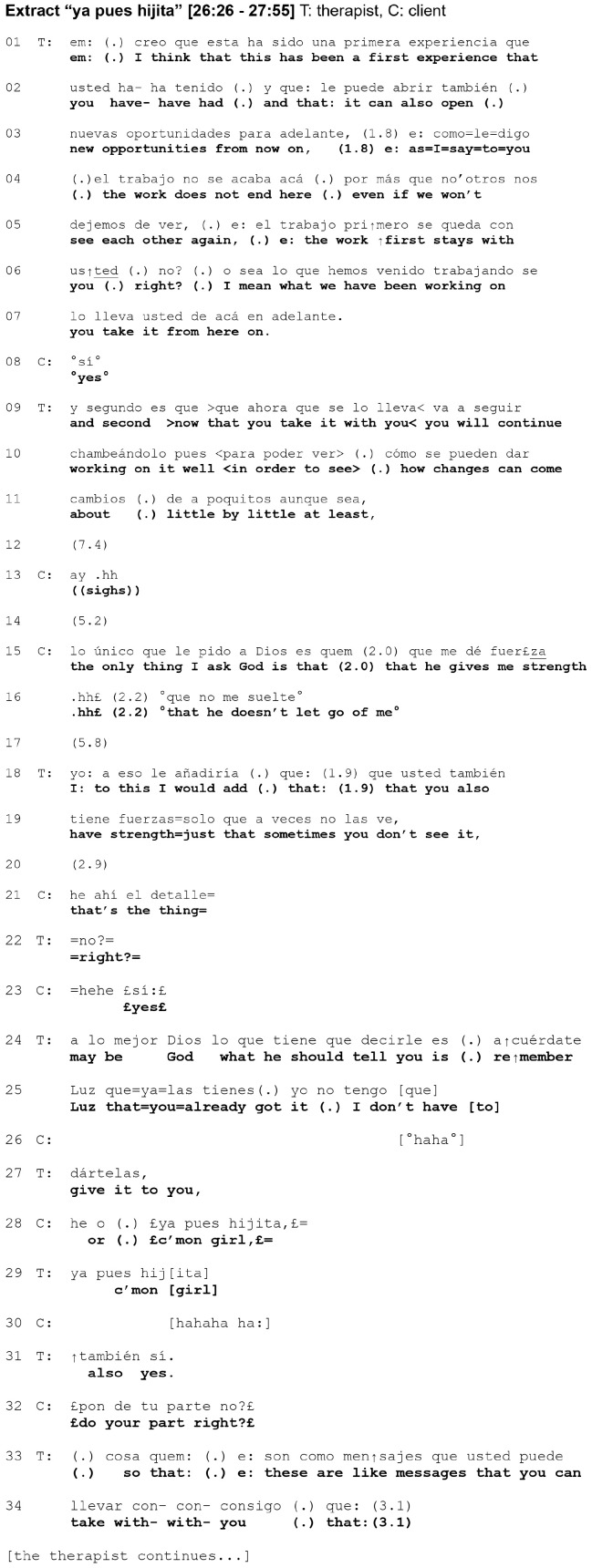


Extending Goodwin’s (2008, 2018) analyses of cooperation and pointing practices to understand psychotherapeutic interaction, Buchholz (2022a) argues that clients have no possibility to point to a perceptual world but only “to conversational objects like topics, experiences or (reported) events” (p. 61). This applies to not only clients but also therapists. The therapist’s interpretation in (18–19) can thus be considered as a pointing action and, accordingly, as an invitation to the client to attend together to her agency within a collaborative participation framework (Goodwin, 1981, 2018). Additionally, the therapist’s invitation shows a distinguishing feature of psychoanalytic therapy: he invites her to “see” something that she sometimes does not “see.” The therapist uses this verb as a “conceptual metaphor” (Lakoff and Johnson, 1980, 1999; Grady, 2007): to see is to know. In this particular case, what is not seen is something the client is unaware of. Moreover, the therapist not only points to the unknown object (agency) but also to the client’s inability to “see” it, that is, to “know” or become aware of it.

Following Peräkylä’s (2019) sequential model, this interpretation by the therapist has the role of an initiating Target Action. Its goal is to solve the challenge to the intersubjective relationship posed by the now moment in section 2 (12–17), which we consider as the Previous Action for this sequence. The interpretation tries to achieve this goal through an “insight” that should lead to the client’s recognition of her own agency. According to prior CA research on psychotherapy, interpretations call for confirmations or disconfirmations by the client (Bercelli et al., 2008; Peräkylä and Antaki, 2008; Peräkylä, 2013). We have pointed out that the therapist’s interpretation can be considered as an invitation to the client to attend together to her agency. Had the client accepted the interpretation, a situation of joint attention would have resulted (Moll and Meltzoff, 2011; Carpenter and Call, 2013), with both therapist and client “seeing” (knowing) the same “thing” (her agency) and also knowing that they were doing so together. This would have brought about a moment of meeting and resolved the crisis.

However, the client’s Response in (21), after a short gap in (20), is ambiguous in this regard and does not seem to express wholehearted agreement. It contains the Spanish colloquial expression “he ahí el detalle,” which is difficult to translate into English. Literally, it means “there is the detail,” but a convenient translation in this context would be “that’s the thing.” Thus, the client does not agree with the interpretation and the joint attention situation is not intersubjectively ratified. Her words “He ahí el detalle” thus acquire a clearer meaning: that is the problem, that I cannot “see” (become aware of) my agency. Had the client confirmed his interpretation, the therapist’s ensuing Third Position Action would have likely closed the sequence by intersubjectively ratifying the alignment of the client’s Response to his Target Action. Instead, however, the therapists’ turn (22) is also ambiguous and does not seem to contain such an intersubjective ratification. This section ends with a turn (23) by the client, in which she laughs openly and says “yes.” It could be seen as affiliative but is also ambiguous.

In summary, our sequential analysis of this section shows that the therapist’s initiating TA in (18–19), the interpretation, fails to bring about a moment of meeting, and so the crisis remains. Nevertheless, we notice that something significant has happened regarding the implicit relational pattern between client and therapist described in section 1 (01–11): for the first time in this episode, the client exercises agency in taking a critical stance toward the therapist.

### Section 4 (24–30)

As previewed above, section 4 (24–30) features a second, successful attempt by the therapist to achieve a moment of meeting, which resolves the challenge posed by the now moment in section 2 (12–17). To examine the interactional unfolding of the moment of meeting we will analyze this section as two overlapping sequences:

**First sequence:** The Previous Action (PA) for the first sequence is the now moment in section 2 (12–17). The initiating Target Action (TA) is the therapist’s turn in (24, 25, 27), the Response (RE) is the client’s turn in (28), and the Third Position Action (TP) is the therapist’s turn in (29).

**Second sequence:** The Previous Action (PA) for the second sequence is the therapist’s turn in (24, 25, 27), the initiating Target Action (TA) is the client’s turn in (28), the Response (RE) is the therapist’s turn in (29), and the Third Position Action (TP) is the client’s turn in (30).

**FIGURE 1:**
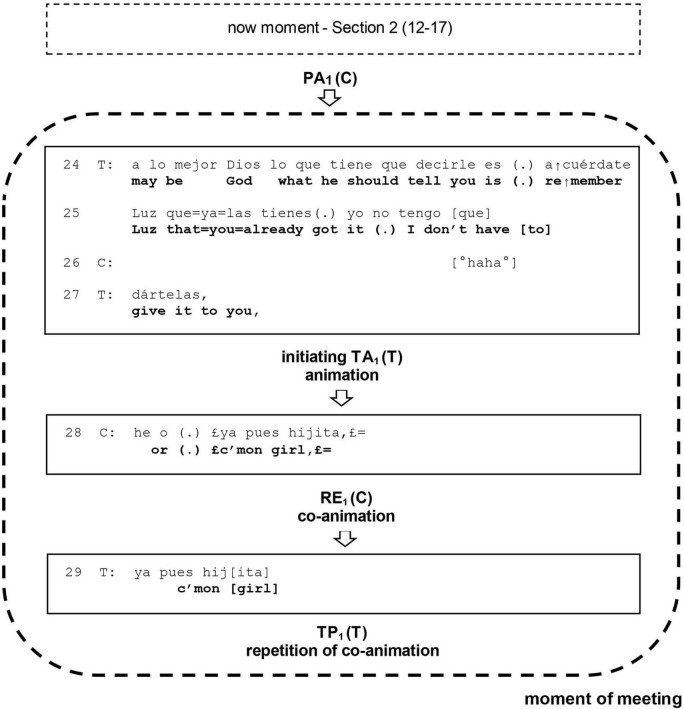
Unfolding of the moment of meeting in section 4 (24–30): first sequence. PA, previous action; TA, target action; RE, response; TP, third position; T, therapist; C, client.

**FIGURE 2:**
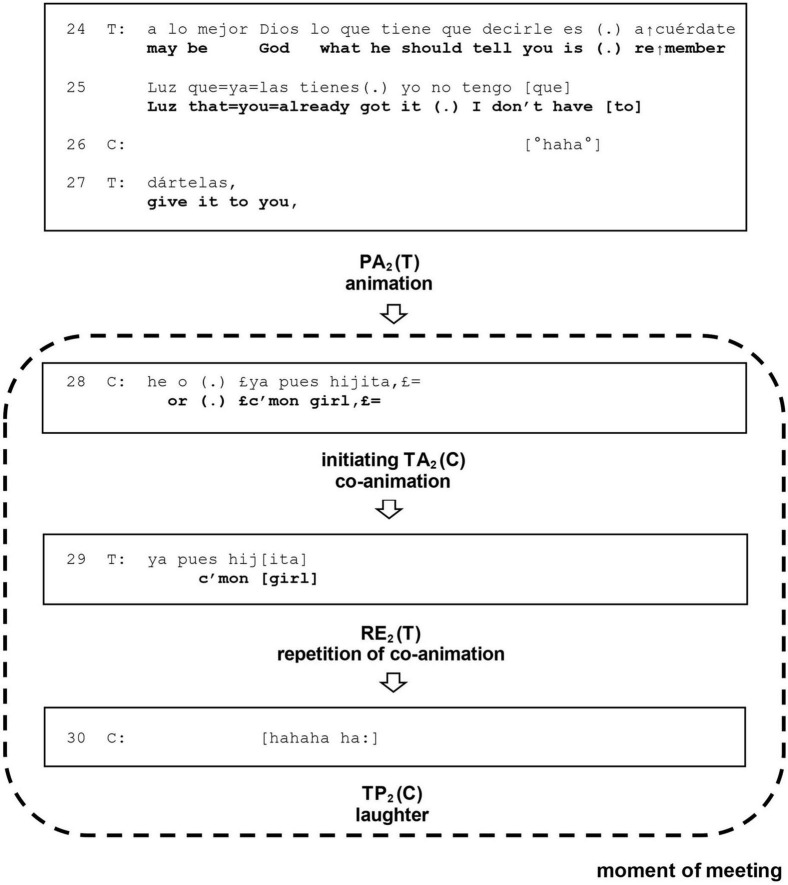
Unfolding of the moment of meeting in section 4 (24–30): second sequence. PA, previous action; TA, target action; RE, response; TP, third position; T, therapist; C, client.

Both sequences share the client’s turn (28). We follow here the fundamental proposal of CA that every contribution in a conversation has both a reactive and an initiating aspect (Deppermann, 1999). Thus, we assume that in its reactive aspect, this turn has the role of Response in the first sequence, and that in its initiating aspect, this same turn has the role of a Target Action in the second sequence. To differentiate both sequences, we will use subscripts _1_ and _2_ for the first and the second sequences, respectively: TA_1_, RE_1_, TP_1_, and TA_2_, RE_2_, TP_2_ (see Figures 1, 2).

### First sequence

This sequence is set in motion by the therapist’s initiating Target Action TA_1_ in his turn (24, 25, 27). It aims to solve the challenge posed to the intersubjective relationship by the now moment in section 2 (12–17), which is the Previous Action PA_1_ for this sequence. It represents a new attempt to achieve a moment of meeting but now through another path. In this turn, the therapist remarkably deviates from his previous interaction style. He resorts playfully to the figure of God, introduced by the client in the now moment in section 2 (12–17), letting him convey to her the content of his former interpretation. We notice three important aspects in this action. First, the therapist steps empathically into the client’s cultural world. In invoking the figure of God, he treats it as part of their common ground, which is clearly affiliative: the emergence of a common ground is one important aspect of “meeting” in psychotherapy (Buchholz, 2022b). Second, in what could be considered a remarkable rhetorical move, he presents God not as an all-powerful external force that should rescue the client but as an external bystander that encourages her to recognize and use her own agency and strength. Third, which is extremely important, he achieves this in the context of a particular practice: animation.

Based on contributions by Goffman (1981) and Clark and Gerrig (1990), Ehmer (2011) defines animation as the embedding of a figure within one’s own speech and simultaneously adopting this figure’s perspective. The figure can be the current speaker, someone else, or an imagined figure, whether human or mythical. Animation is the demonstration of the figure’s (speech-) action in a mental space, and thus makes us directly experience the depicted aspects of the animated speech. Moreover, this animation by the therapist clearly shows the characteristics attributed by Goodwin (2018) to cooperative action: the “process of building something new through decomposition and reuse with transformation of resources placed in a public environment by an earlier actor” (p. 3). Thus, taking the figure of God previously introduced by the client in the now moment, the therapist transforms it to create something new in the form of animation.

In her study on trouble-talk, Cantarutti (2022) shows that tellers use animation of their own affective reactions to experiences in order to cast themselves as victims and the recipients of their narration as witnesses. Consequently, the therapist’s practice of animation introduces a moment of intense emotion, affiliation, closeness, and intimacy. The client reacts to this playful and affiliative move with a laugh in (26), which overlaps with the last TCU of the therapist’s turn. This laugh can be seen as a reaction to the playful animation and an expression of surprise and joy at this different way-of-being-with-her by the therapist, who is now warm, intimate, and playful, in contrast to his previous, rather distant and formal demeanor (Vásquez-Torres, 2021).

In her Response RE_1_ in (28), the client reacts in a strongly affiliative way; however, instead of merely agreeing with the stance of the therapist conveyed in this playful way, she also animates God herself, turning the practice of animation of the therapist into a shared practice of co-animation. Animation is a relevant practice for not only tellers but also recipients. Thus, recipients often offer, in a contiguous position to the teller’s animation, a responding co-animation of the same figure, thereby validating and amplifying the teller’s affective display (Cantarutti, 2022). Co-animation turns the first speaker’s experience into a common cause. Consequently, the complementary practices of animation by the therapist and co-animation by the client result in a moment of intense affiliation.

The client does not confine her co-animation to repeating the words of the therapist but in her incrementation she puts new words into God’s mouth, sending herself an invigorating message of encouragement and admonition. This wakeup call—“¡ Ya pues hijita!”—can be translated as “c’mon girl!” in English. On the one hand, the client thereby appropriates the stance expressed by the therapist’s interpretation in (18–19), finally affiliating with him. On the other hand, in her animation she uses a colloquial and more familiar expression, recycling the very words her own brother said to her on a previous occasion. Her laugh in (28) seems to be a continuation of her previous laugh in (26), and helps to introduce the co-animation with a powerful and humorous message of self-encouragement. This may contribute to framing the playful scenario and display of intimacy (Glenn, 2003).

Thus, the client’s co-animation in her Response RE_1_ in (28) meets the therapist’s playful animation in (18–19). In this way, the complementary practices of animation by the therapist and co-animation by the client prompt a moment of intense affiliation: a moment of meeting. In his next turn in (29), the Third Position Action TP_1_ action that closes this first sequence, the therapist repeats the client’s words “Ya pues hijita” (“c’mon girl”). The practice of repetition in this context is highly affiliative, so this move strengthens the affiliation elicited by the therapist’s animation and the client’s co-animation. Through his repetition of the client’s co-animation, the therapist closes this first sequence by intersubjectively ratifying the moment of meeting in third position.

### Second sequence

This sequence is set in motion by the initiating aspect of the client’s co-animation in (28), which constitutes the Target Action TA_2_. Accordingly, the therapist’s animation in (24, 25, 27) is the Previous Action PA_2_ for this sequence. The therapist’s repetition of the client’s co-animation in (29) is the Response RE_2_, which “meets” the client’s initiating Target Action TA_2_. This meeting of actions in the second sequence also prompts a moment of intense affiliation: a moment of meeting. The loud and joyful laugh of the client in (30) is the Third Position Action TP_2_, which closes this second sequence, intersubjectively ratifying the moment of meeting in third position.

We claim that in and through these two overlapping sequences in section 4 (24–30), therapist and client achieve a moment of meeting. As our analysis reveals, it is “a present moment in which the two parties achieve an intersubjective meeting” (Stern, 2004, p. 151), a moment where “Intersubjective ‘fittedness’ is sought,” where both “share an experience and they know it implicitly” (Stern, 2004, p. 168). In this moment of meeting, the client and therapist finally solve the challenge posed to the intersubjective relationship by the now moment in section 2 (12–17).

What prompted this moment of meeting to occur at this point of the exchange? At first sight, we might think it is mainly a consequence of the therapist’s initiating Target Action TA_1_ in the first overlapping sequence, whereby he introduced the animation of God in (24, 25, 26). This turn clearly meets Stern’s criteria for a therapist’s contribution that should be able to bring about a moment of meeting, namely “an authentic response finely matched to the momentary local situation,” that “must be spontaneous and must carry the therapist’s personal signature,” reaching “beyond a neutral, technical response” (Stern, 2004, p. 168). However, our analysis applying CA and particularly Peräkylä’s (2019) model of sequential organization of psychotherapy allows us to give a more complex response to this question. On the one hand, the moment of meeting is prompted by the therapist’s initiating Target Action TA_1_ in the first sequence. On the other hand, both the client’s initiating Target Action TA_2_ and the therapist’s Response RE_2_ in the second, overlapping sequence play a crucial role in the moment of meeting. Thus, in (28) the client turns the animation practice introduced by the therapist into a shared practice of co-animation, and the therapist reacts in (29) with a repetition of the client’s co-animation through which he both aligns and affiliates with her. This is the point of the exchange where the moment of meeting comes about.

We also notice in this second sequence a significant change from how the client–therapist interaction has been unfolding up to this point. On the one hand, the therapist displays through the design of his Response RE_2_ that they are jointly engaged in the same interactional project of co-animation. On the other hand, he adopts the more colloquial, familiar expression introduced by the client, thereby granting her an initiating role in this sequence. This markedly contrasts with their previous interaction in most of the session, especially in section 1 (01–11), with the therapist dominating the exchange and talking in a didactic style to the client, who limited herself to giving weak signals of acknowledgment. In both sequences in section 4 (24–30), both participants contribute actively to the exchange, alternatively proposing an initiating action or following the other’s initiating action. Thus, a momentary but significant transformation in the here-and-now relationship between client and therapist comes about, manifested in their interaction: the client now exercises her own agency to assume an active role, which the therapist ratifies.

Accordingly, the occurrence of this moment of meeting is not just the consequence of a remarkable contribution by the therapist in (24, 25, 27): the moment of meeting emerges from the interaction process of therapist and client, and is thus co-created or co-constructed (Ugarte, 2019). As Stern (2004) comments, “A moment of meeting is a special case of ‘doing something together”’ (p. 176). The mutuality displayed here can be seen as a practice of “doing We” (Buchholz, 2022a,b). The client’s joyful laughter in (30) can also be interpreted as an affective expression of this moment of playful co-creation, of having done something together, and of the joy and surprise of being in this new and different place in relation with another.

It is perhaps the therapist’s Response RE_2_ in the second sequence, (29), even more than his initiating Target Action TA_1_ in the first sequence, (24, 25, 27), that best meets Stern (2004) criteria for a contribution able to bring about a moment of meeting. This is because that Response is an authentic, spontaneous, and personal contribution, reaching beyond a neutral and technical intervention, and is especially finely tailored to the local situation. This turn of the therapist enables the co-creation or co-construction of the moment of meeting and, therefore, fosters the client’s agency. It is very significant that this contribution by the therapist, which should be considered the most “therapeutic” in the whole exchange, is not in an initiating position as Target Action TA_1_ but in a reacting position as Response RE_2_ to the client’s initiating Target Action TA_2_.

As we have seen before, through pointing to the client’s agency in his interpretation in section 3 (18–23), the therapist invited the client to enter a framework of joint attention, but his attempt was unsuccessful. Subsequently, in section 4 (24–30), the therapist introduces the animation in (24, 25, 27) and in this context uses the figure of God to point to the client’s agency again. Interestingly, the client’s Response RE_1_ in (28) is not an intersubjective ratification of the pointing through recognition of her agency in a joint attention framework. Instead, her response displays that she takes the therapist’s animation as an invitation to enter a different participation framework (Goodwin, 1981, 2018), namely the playful space of animation, where she exercises that very agency in her interaction with him.

### Section 5 (31–34)

Although the therapist’s next turn in (31) is affiliative, through its design he retreats from his more playful and personal interaction style in section 4 (24–30) to a more distanced one. The client’s turn in (32) can be seen as both an attempt to continue with the playful co-animation and an elaboration of the therapist’s interpretation. In his next turn in (33–34), the therapist does not respond to the playfulness. The moment of meeting thus ends. However, the therapist does ratify their mutual agreement regarding the content of his interpretation of the client’s agency, which he now reformulates as “messages that you can take with you,” clearly alluding to the message of encouragement in the co-animation.

We have presented the results of our single-case analysis using CA, particularly Peräkylä’s (2019) sequential model, to illustrate how the interactional unfolding of a momentary transformation in the client–therapist here-and-now relationship comes about, as manifested in their interaction. We have shown that this momentary transformation of relation corresponds to a moment of meeting, which resolves a challenge to the intersubjective relationship posed by a now moment (Stern, 2004). Next, we will discuss some theoretical implications of our results.

## Discussion

Many researchers and clinicians would likely agree that this episode contains a therapeutic change, even if only momentary. What makes this episode therapeutic? We can point to the client’s acceptance of the therapist’s interpretation in (18–19), which explicitly aims at the client recognizing her own agency. From a more traditional perspective on the effects of psychoanalytic therapy, this interpretation is arguably therapeutic because it gives the client an “insight” into her subjective mental life that can bring about changes in representations about herself and her relations to others (Groeben et al., 1988; Krause, 2005).

From a more relational perspective, however, we claim that another significant therapeutic event occurs in this segment. Thanks to the sequentially accomplished shared practice of co-animation in (28–29) a momentary but significant change occurs in the here-and-now relationship between client and therapist. Specifically, the client breaks out of the passive role assumed previously and takes an active role in the interaction, which is then ratified by the therapist. Thus, in this sequence, the client exercises her own agency in interacting with the therapist, thereby enacting the very content of the therapist’s interpretation that she has agency and strength in the here-and-now exchange between them. We witness a momentary change in the client’s way-of-being-with-another, or a transformation of relation, that emerges in this sequence. Moreover, it is plausible that the client’s acceptance of the therapist’s interpretation and the relational event reinforce each other: the therapist’s animation makes it possible for the client to affiliate with his stance in the interpretation, while the co-animation sequence brings about the change in the relational pattern.

Key aspects of transformation of relation that are investigated by CA research on psychotherapy are *agreement* and *disagreement* or *resistance*, *affiliation* and *disaffiliation*, and the *epistemic relation* (Peräkylä, 2019). One important goal of our paper has been to draw attention to an additional, significant aspect of transformation of relation that can be investigated in this field: the transitory emergence of new forms of relatedness in and through sequentially organized talk and action in psychotherapy.

Our sequential analysis applying CA has shown that the moment of meeting in our segment is interactionally accomplished through speaking practices that foster affiliation and alignment (Voutilainen et al., 2010; Muntigl et al., 2012; Lindström and Sorjonen, 2013; Scarvaglieri, 2020; Guxholli et al., 2021; Peräkylä and Buchholz, 2021). We can describe it as an occasion of heightened emotional intimacy in the interaction, characterized by participants’ mutual display of affective attunement to each other (affiliation) and by the disposition of each to “go along with” the other’s suggested courses of action (alignment). Another crucial feature of the sequential unfolding of this moment of meeting is the significant role of humor, laughter, and playfulness (Vásquez-Torres, 2021). CA research has shown the importance of humor and laughter in psychotherapeutic interaction (Valentine and Gabbard, 2014; Diogini and Canestrari, 2018). Humor, laughter, and playfulness are thus important ingredients and expressions of the transformation of relation occurring in our segment.

One main conclusion of our analysis is that this moment of meeting does not result from a single contribution by the therapist but emerges sequentially in the interaction between therapist and client, to which both equally contribute: it is co-created or co-constructed (Ugarte, 2019; Durand, 2023). Using a metaphor introduced by the BCPSG (Stern, 2009), the sequence leading to the moment of meeting in our segment can be compared with a dance, where therapist and client found their own rhythm and own way to move along together during the therapeutic process. In that regard, moments of meeting represent a form of what Buchholz (2022a,b) calls “doing We”: “Psychotherapy cures by perceiving and being perceived. Like in a mother-baby relationship. This mutuality is a practice of ‘doing We’; it is done by observable practices and nevertheless it establishes mind-meeting” (Buchholz, 2022b, p. 321).

A last issue we should address is the role that Stern (2004) attributes to language in moments of meeting. Two passages of his influential book can help us to clarify his view on that issue. In one passage, where he presents a moment of meeting between two persons, not in psychotherapy but in a real life setting, he states: “Once they start talking, they will also act along with the words –small movements of face, hands, head, posture. These accompany, follow, or precede the words. The explicit then becomes the background for the implicit momentarily” (Stern, 2004, p. 175). He further states: “These relational moves are enacted out of consciousness, leading up to the moment of meeting—their hands move to meet” (Stern, 2004, p. 175). In another passage he writes: “It is important to remember that the experience contained in present moments is occurring in parallel with the exchange of language during a session. The two support and influence each other in turns. I am not trying to lessen the importance of language and the explicit in favor of implicit experience. I am trying to call attention to direct and implicit experience because it has been relatively neglected” (Stern, 2004, p. 222). Because moments of meeting are intersubjective present moments, this statement applies to them as well.

We note that in these two passages Stern identifies language with the explicit, i.e., what is communicated directly in the semantic content of the linguistic expressions (words and sentences) exchanged by therapist and client. Because in his view the sequence of relational acts that leads to a moment of meeting occurs at an implicit level, it should be parallel to the exchange of language at the explicit level. Interestingly, although some of his examples, such as the two-handed shake, do not imply words at all, other examples, such as the client suddenly facing the therapist, involve the exchange of verbal utterances. Nonetheless, in his theoretical account, Stern (2004) does not consider the possibility that the exchange of language itself can lead to a moment of meeting. The analysis of our segment applying CA, particularly Peräkylä’s (2019) sequential model, suggests a more nuanced view of that issue. The co-animation sequence by which the moment of meeting is brought about is made up of strings of words, i.e., of verbal utterances. It is difficult to imagine how therapist and client could have carried out this sequence without an exchange of language.

This raises a crucial question: how can an exchange of language, which belongs to the explicit level, lead to a moment of meeting, that should be the result of acts that take place at the implicit level? The answer to this question is provided by a key assumption of CA (Wilkinson and Kitzinger, 2008), which is also a major contribution of linguistic pragmatics, particularly speech acts theory: talk is a form of action, we can do things with words (Austin, 1962; Collavin, 2011). Every time speakers emit a verbal utterance in a particular context, they thereby perform an action. Therefore, the co-animation sequence in our segment is brought about by the sequential exchange of actions performed through the production of verbal utterances by client and therapist. Drawing on Heritage’s (1984) influential formula, we claim that this moment of meeting is *talked into being* through that exchange. Consequently, the moment of meeting in our segment occurs not *in parallel* with the exchange of linguistic utterances between client and therapist. It occurs *through* the exchange of such linguistic utterances and *through* the sequence of actions carried out by that exchange. It does not result from a sequence of actions that takes place *along with* the words, but from a sequence of actions that are carried out *through* the words uttered by client and therapist in the exchange.

However, that sequence of actions does not take place at an explicit level, because it occurs without the therapist explicitly addressing it in the content of an interpretation. This is consistent with Stern’s observation that “The moment of meeting need not be verbalized to effectuate change” (Stern, 2004, p. 220). Thus, a further significant outcome of our analysis is that, parallel to the sequential exchange of verbal utterances at the explicit level, the sequential exchange of actions performed by those verbal utterances occurs at the implicit level. It is at this implicit dimension of verbal interaction that the transformation of relation in the moment of meeting occurs. In that regard, in the last page of his book, Stern makes a remarkable comment about the role of verbal meaning making and narrativizing in talking therapies, which is very close to Buchholz’s (2022a) approach on mutuality and “doing We” in psychotherapeutic interaction. Stern states that these verbal activities, which can bring about therapeutic change, can also be a vehicle by which client and therapist *do something together*: “It is the doing-together that enriches experience and brings about change in ways-of-being-with-others through the implicit processes discussed” (Stern, 2004, p. 227). The analysis of our segment reveals that this view of Stern’s should be extended beyond the specific “therapeutic” verbal activities of meaning making and narrativizing. It applies to any verbal interaction between client and therapist in which the sequential *doing-together with words* leads to a moment of meeting, bringing about change, at least momentarily, in the implicit ways-of-being-with-others of the client.

## Limitations and future directions

One important limitation of our study is that we have access only to the audio recording of the session. An integration of aspects of visual para-verbal and non-verbal interaction would be useful to achieve a more comprehensive analysis of such episodes.

This has been a single case study. The next step in the research should be to build a collection of such episodes in therapeutic interaction in order to find common interactional features between them.

As we have pointed out, the change in the here-and-now relationship between client and therapist that we observe in our segment is momentary. It would be important as well to examine if such changes take place during a whole session and during the course of various sessions from a complete therapeutic process.

The methods of CA do not allow us to correlate such momentary transformations in the relational pattern between client and therapist with long-lasting changes in the relational pattern and in the emotional well-being of the client during and after the treatment. Investigations that link CA with other methods in the field of psychotherapy research would be helpful in attaining this goal.

Clinicians would surely agree that not every session in a psychotherapeutic process contains salient interpersonal events like the moment of meeting we have analyzed. This does not mean that changes prompted by interpersonal events cannot occur in such sessions. In that regard, Stern (2004) observes that more spectacular interpersonal events like now moments or moments or meeting are unusual, but that progressive changes can also take place gradually through less charged interpersonal moments. An analysis based on CA, like the one we have presented in this paper, can also contribute to the understanding of such moments and of the gradual changes they can bring about.

## Data availability statement

All data supporting the conclusions of this study are included in this article/Supplementary material, further inquiries can be directed to the corresponding author.

## Ethics statement

The studies involving humans were approved by the Comité de Ética de la Investigación para Ciencias Sociales, Humanas y Artes, Vicerrectorado de Investigación, Pontificia Universidad Católica del Perú (Pontifical Catholic University of Peru). The studies were conducted in accordance with the local legislation and institutional requirements. The participants provided their written informed consent to participate in this study. Written informed consent was obtained from the individual(s) for the publication of any potentially identifiable images or data included in this article.

## Author contributions

All authors have participated in the five steps of the research described in the section Data and methods, contributed to the manuscript, and approved the submitted version.
