# Supplementary material for: Doing-together with words: the sequential unfolding of a moment of meeting in a psychoanalytic therapy session
Source: Front Psychol. 2023 Dec 7;14:1205500. doi: 10.3389/fpsyg.2023.1205500 (PMC10748479; doi:10.3389/fpsyg.2023.1205500)
Supplement: Supplementary file 1 [file Table_1.pdf]

APPENDIX: CA TRANSCRIPTION CONVENTIONS

(Adapted from: Jefferson, 2004; Ten Have, 2007; Raymond and Olguín, 2022)

T, C:	Speaker identification. T: therapist, C: client.
re[mem]ber<br>[word]	Onset and offset of overlapping talk
(.)	Pause less than 0.2 seconds
(2.6)	Timed pause
=	Latching: no gap between utterances
.	Falling final intonation
?	Rising final intonation
,	Slightly rising final intonation
_	Level final intonation
REMEMBER	Talk is louder than the surrounding talk
°remember°	Talk with lower volume than the surrounding talk
remember	stress or emphasis
>remember<	Faster-paced talk than the surrounding talk
<remember>	Slower-paced talk than the surrounding talk
reme:mber	Prolongation of the immediately preceding sound
remem-	abrupt cutoff
£remember£	smiley voice
#remember#	‘creaky’ voice
.hh	aspiration
hh	expiration
hehe, haha	laughter
((coughs))	non-verbal behaviour
